# Case report and literature review: histologic transformation from EGFR-mutant lung adenocarcinoma to squamous cell carcinoma with newly detected SMAD4 and ATM variants

**DOI:** 10.3389/fonc.2026.1982968

**Published:** 2026-10-27

**Authors:** Chao Wang, Shuo Wang, Daixi Wu, Yanni Lou, HongYan Liu, Yu Tang, Fei Su, Liqun Jia

**Affiliations:** 1 Department of Integrative Oncology, China-Japan Friendship Hospital, Beijing, China; 2 Deaprtment of Oncology, Beijing Hospital of Traditional Chinese Medicine, Capital Medical University, Beijing, China; 3 Department of Pathology, China-Japan Friendship Hospital, Beijing, China; 4 Department of Oncology, Beijing Chaoyang District Hospital of Traditional Chinese Medicine, Beijing, China

**Keywords:** adenocarcinoma-to-squamous cell transdifferentiation, ATM, EGFR-TKI resistance, lineage plasticity, SMAD4

## Abstract

**Background:**

Adenocarcinoma-to-squamous cell carcinoma transdifferentiation (AST) is an uncommon but clinically important form of histologic evolution in EGFR-mutant lung adenocarcinoma. Its underlying molecular mechanisms remain incompletely understood. Longitudinal genomic profiling may help characterize molecular alterations accompanying lineage transdifferentiation.

**Case presentation:**

A 60-year-old woman with lung adenocarcinoma previously underwent surgical resection, and molecular testing identified an EGFR exon 19 deletion (EGFR 19del). After multiple lines of EGFR tyrosine kinase inhibitors and systemic therapy, she developed pleural recurrence. Repeat biopsy revealed a squamous phenotype, with positive p40 and CK5/6 staining and loss of TTF-1 and Napsin A expression, supporting AST. Longitudinal next-generation sequencing (NGS) showed that EGFR 19del and a TP53 p.R175H mutation were present both at baseline and after transformation, whereas SMAD4 p.D351V and ATM p.E1139K variants were newly detected only in the post-transformation tumor. After transformation, the patient received carboplatin, nab-paclitaxel, and tislelizumab and achieved a partial response.

**Conclusion:**

We report a case of AST after multiple lines of therapy in a patient with EGFR-mutant lung adenocarcinoma and summarize potential molecular mechanisms and treatment developments in AST. Longitudinal NGS identified SMAD4 p.D351V and ATM p.E1139K variants in the post-transformation tumor. Functional evidence suggests that SMAD4 p.D351V may impair SMAD4-dependent BMP/TGF-β signaling, whereas the functional significance of ATM p.E1139K remains unclear. Further studies are needed to determine whether either variant contributes to AST.

## 1 Introduction

Although EGFR tyrosine kinase inhibitors (EGFR-TKIs) are effective in EGFR-mutant non-small cell lung cancer (NSCLC), acquired resistance commonly develops after approximately 10–14 months of treatment (1). Established resistance mechanisms include the EGFR T790M missense mutation, MET or HER2 amplification, epithelial–mesenchymal transition, and histologic transformation (HT) (1). HT is an uncommon but challenging mechanism of resistance to EGFR-TKIs. Among patients who underwent repeat biopsy after EGFR-TKI resistance and had a confirmed histologic diagnosis, HT occurred in approximately 2.8% (74/2,624). Transformation from adenocarcinoma to small-cell lung cancer (SCLC) was the most common form, accounting for 65% (48/74) of HT cases, followed by adenocarcinoma-to-squamous cell carcinoma transdifferentiation (AST), which accounted for 15% (11/74) (2). Among patients with EGFR-mutant lung adenocarcinoma resistant to osimertinib, the reported incidence of AST is 5.5%–9% (3, 4). One retrospective study found numerically shorter overall survival in patients with HT than in those without transformation, although the difference was not statistically significant (30.8 vs. 41.2 months from TKI initiation and 6.6 vs. 12.9 months from transformation, respectively) (4). Although relatively rare, AST indicates substantial histologic and biological evolution of the tumor and complicates subsequent treatment decisions. The molecular evolution and underlying mechanisms of AST remain incompletely characterized, and optimal treatment strategies after transformation have yet to be established. Here, we report a patient with EGFR 19del-positive lung adenocarcinoma who developed disease progression after multiple lines of EGFR-TKIs, chemotherapy, and immunotherapy, with repeat biopsy and immunohistochemistry confirming a squamous phenotype. We also discuss molecular changes observed by longitudinal NGS and briefly review potential mechanisms and treatment developments in AST.

## 2 Case presentation

A 60-year-old woman who had never smoked underwent lung surgery in February 2019. Pathologic examination showed acinar-predominant invasive adenocarcinoma of the right lower lobe. Immunohistochemistry was positive for TTF-1 and Napsin A, and the tumor was staged as pT1cN0M0, stageIA3. NGS of the tumor using a targeted 50-gene panel identified EGFR 19del and a TP53 p.R175H mutation. Pleural metastases developed in October 2021, and first-line osimertinib was initiated at 80 mg once daily. In October 2024, follow-up CT showed right pleural thickening and a right pleural effusion, raising concern for disease progression; treatment was changed to second-line aumolertinib (110 mg once daily). PET/CT in January 2025 showed diffuse right pleural thickening and hypermetabolic right hilar lymph nodes, both considered metastatic. Plasma-based targeted NGS using a 680-gene panel did not identify clinically relevant variants. Given disease progression and resistance to EGFR-TKIs, third-line treatment with pemetrexed, nedaplatin, bevacizumab, and sintilimab was initiated in February 2025, followed by maintenance sintilimab with or without bevacizumab. PET/CT in May 2026 showed metastases involving the right pleura, peritoneum, thoracic and abdominal lymph nodes, and bone (**Figure 1**). Because of continued disease progression, a percutaneous lung biopsy was performed and showed squamous cell carcinoma. Immunohistochemistry was positive for CK5/6 and p40 and negative for TTF-1; p53 showed diffuse strong nuclear staining, consistent with a TP53 missense mutation (**Figure 2**). Tissue NGS with matched peripheral blood as a control retained the original EGFR 19del and TP53 p.R175H mutation and identified additional variants in SMAD4 (c.1052A>T, p.D351V; variant allele frequency [VAF], 15.79%) and ATM (c.3415G>A, p.E1139K; VAF, 4.97%). Both were classified as class III variants of uncertain significance under the American College of Medical Genetics and Genomics (ACMG) criteria. The PD-L1 tumor proportion score (TPS) was <1%. In May 2026, fourth-line treatment with nab-paclitaxel, carboplatin, and tislelizumab was initiated. Because the bone and peritoneal metastases were nonmeasurable lesions and the short-axis diameters of thoracic and abdominal lymph nodes were <1.5 cm, they did not meet the criteria for target lesions; therefore, response assessment was based primarily on the pleural lesion. Follow-up CT in October 2026 showed a reduction in the pleural lesion, corresponding to a partial response (PR) according to RECIST version 1.1 (40.3% reduction; **Figure 3**). During fourth-line treatment, no clinically significant adverse events or immune-related adverse events were observed. The patient had a history of hypothyroidism for 11 years, which was well controlled, and no history of cardiovascular or cerebrovascular disease or other chronic illnesses. The clinical course is summarized in **Figure 4**.

**Figure 1:**
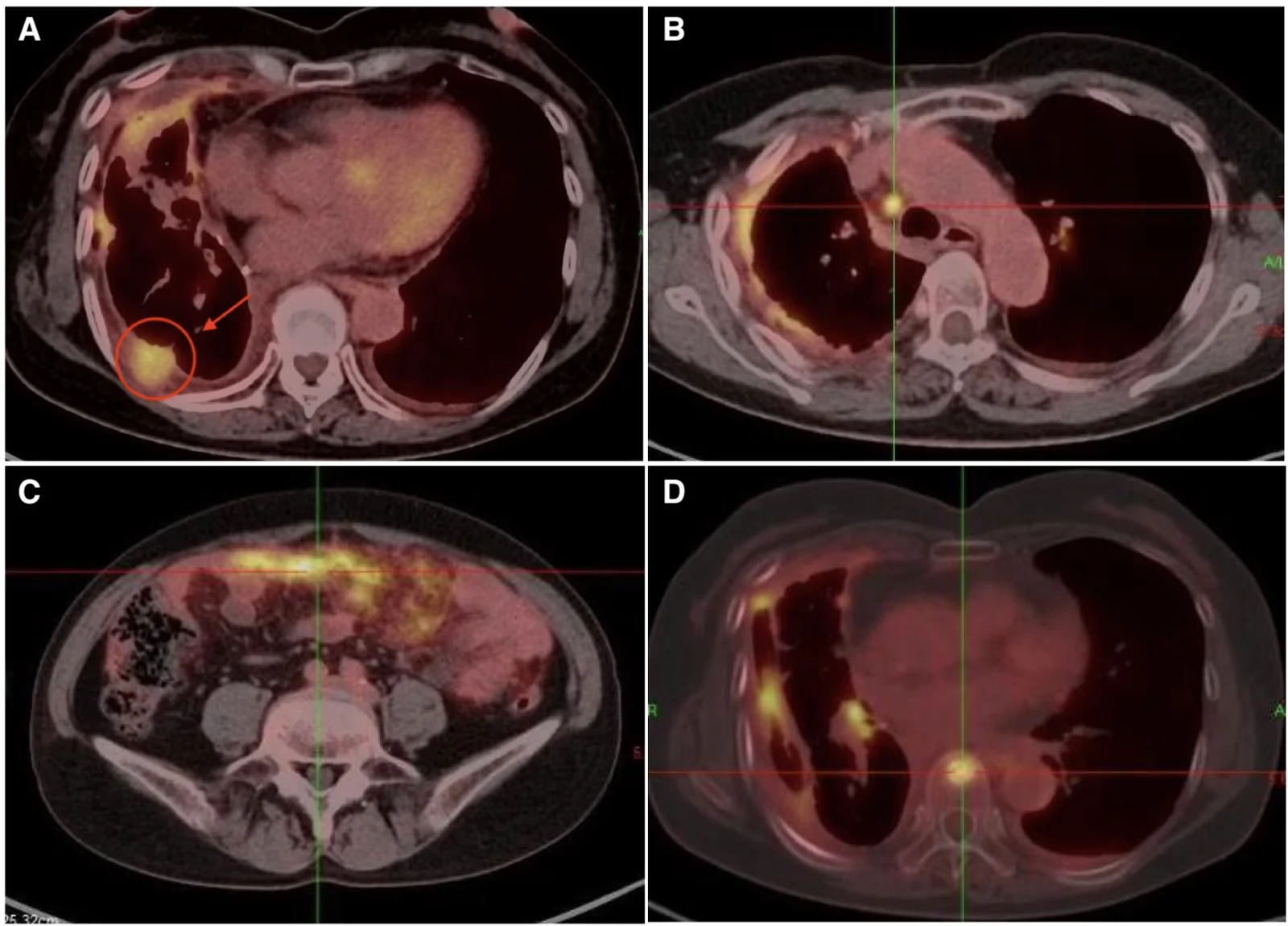
PET/CT imaging in May 2026. **(A)** Increased FDG uptake in the right pleura (SUVmax = 5.9); **(B)** Hypermetabolic mediastinal lymph nodes (SUVmax = 11.7); **(C)** Increased FDG uptake in the peritoneum (SUVmax = 10.5); **(D)** Increased FDG uptake in the vertebral body (SUVmax = 8.6).

**Figure 2:**
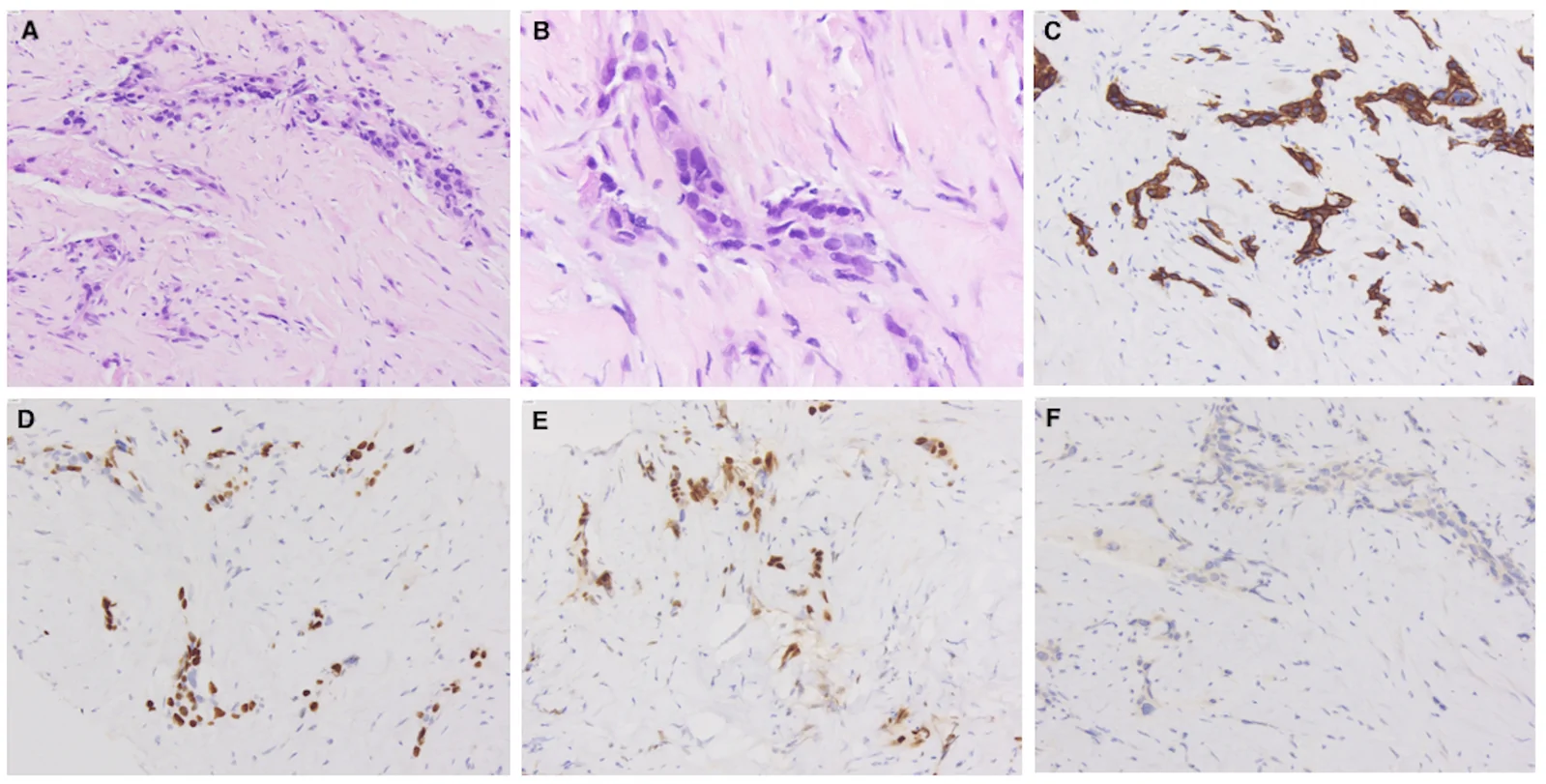
Pathologic findings from percutaneous lung re-biopsy in May 2026. **(A, B)** Hematoxylin and eosin staining showing poorly differentiated carcinoma without obvious glandular structures, keratin pearls, or intercellular bridges (magnification: A, ×200; B, ×400). **(C, D)** Immunohistochemical staining for CK5/6 and p40, respectively, showing diffuse, strong CK5/6 positivity in the cytoplasm/membrane and nuclear p40 positivity, consistent with a squamous phenotype. **(E)** p53 immunohistochemistry showing diffuse, strong nuclear staining in tumor cells, consistent with a TP53 missense mutation (×200). **(F)** TTF-1 immunohistochemistry showing absence of nuclear staining in tumor cells (×200).

**Figure 3:**
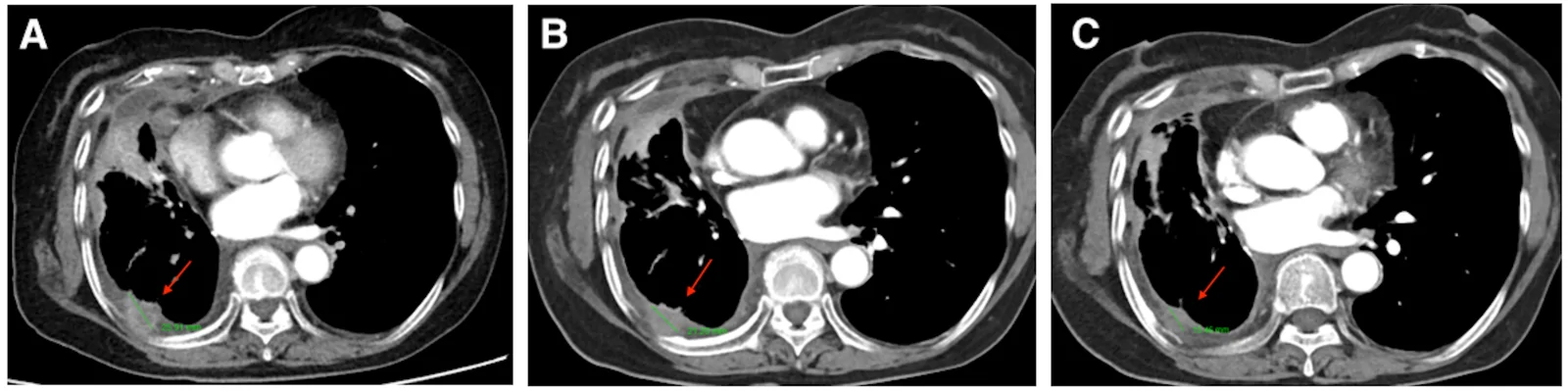
Comparison of contrast-enhanced chest CT images before and after treatment following AST. **(A)** Contrast-enhanced chest CT on May 9, 2026, showing the pleural lesion indicated by the arrow (25.91 mm). **(B)** Contrast-enhanced chest CT on July 8, 2026, showing a reduction in the size of the pleural lesion indicated by the arrow compared with baseline (21.20 mm; 18.2% reduction). **(C)** Contrast-enhanced chest CT in October 2026, showing a further reduction in the size of the pleural lesion indicated by the arrow compared with baseline (15.46 mm; 40.3% reduction).

**Figure 4:**
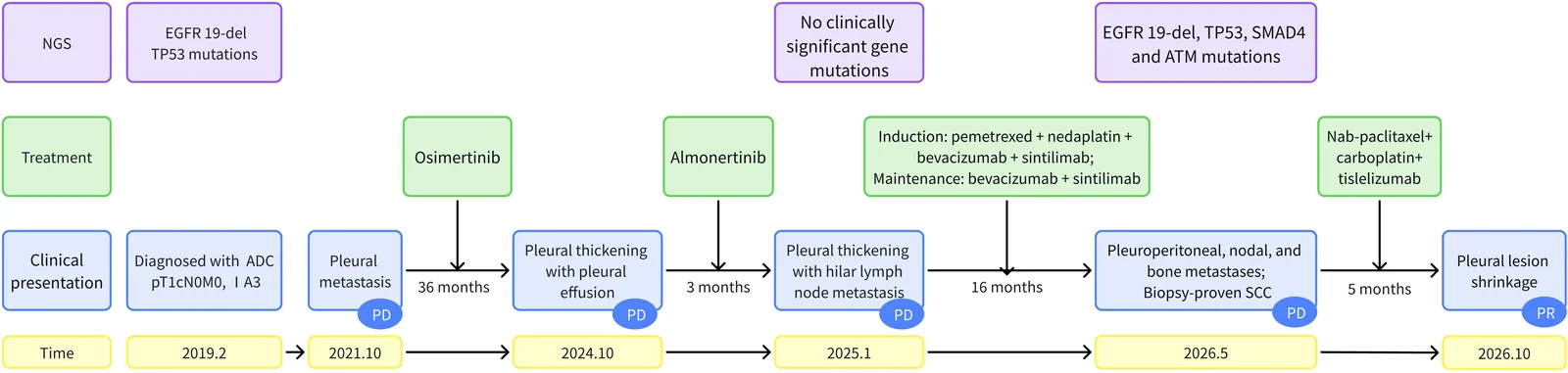
Treatment timeline.

## 3 Discussion

### 3.1 Mechanisms of histologic transformation

Three explanations have been proposed for the detection of squamous cell carcinoma (SCC) on repeat biopsy in EGFR-mutant lung adenocarcinoma resistant to EGFR-TKIs: selection of a pre-existing squamous subclone, a second primary tumor, or true lineage transdifferentiation (5). In this case, the primary tumor had been surgically resected at initial diagnosis, and the original pathology assessment did not report an SCC or adenosquamous carcinoma component. Although a large surgical specimen allows more comprehensive assessment of tumor morphology than a small biopsy, a minor unsampled squamous component cannot be completely excluded. The transformed tumor retained EGFR 19del and the TP53 p.R175H mutation, suggesting a shared clonal origin; however, these findings cannot distinguish lineage transdifferentiation from selective expansion of a pre-existing squamous subclone. Because the original surgical slides were unavailable for re-review (the surgery was performed at another hospital), this distinction could not be resolved definitively. Nevertheless, the tumor was initially diagnosed as adenocarcinoma and subsequently showed a clear squamous phenotype, making the clinical and pathologic course consistent with AST.

The mechanisms underlying AST remain incompletely understood. Available evidence implicates loss of LKB1/STK11, activation of the PI3K/AKT/mTOR pathway, and dysregulation of transcription factors. Loss of LKB1/STK11 is among the better-characterized molecular mechanisms associated with AST and links metabolic and redox alterations to suppression of adenocarcinoma lineage factors and activation of squamous programs (6–9). However, no LKB1/STK11 alteration was detected in the samples tested in this case, limiting the direct relevance of this mechanism to the present patient. Aberrant PI3K/AKT/mTOR and MYC signaling has also been associated with acquisition of squamous lineage features in preclinical and clinical AST models (3, 10, 11). Lineage-defining transcription factors and epigenetic regulators also contribute to AST. Loss of NKX2–1 together with SOX2 activation can favor a squamous lineage program, while alterations involving TET2, and the RB pathway have been linked to lineage plasticity and squamous transformation (12–14). These findings highlight the broader role of transcriptional and epigenetic reprogramming in AST.

In this patient, SMAD4 (c.1052A>T, p.D351V; VAF, 15.79%) and ATM (c.3415G>A, p.E1139K; VAF, 4.97%) variants were detected in the post-transformation tumor. Notably, both genes were included in the 50-gene panel used at initial diagnosis, but neither variant was detected at that time. These variants were therefore newly detected in the post-transformation sample; however, it remains uncertain whether they were truly acquired during the transformation process.

Most studies investigating SMAD4 function have used genetic engineering approaches to conditionally delete or knock out SMAD4, and these studies suggest that SMAD4 loss plays an important role in the initiation and progression of SCC. SMAD4 loss can directly affect epithelial cells, allowing them to escape TGF-β-mediated growth inhibition, promoting cancer stem-cell expansion, and increasing genomic instability, thereby facilitating SCC initiation. In addition, SMAD4-deficient epithelial cells overexpress and release TGF-β into the tumor microenvironment, inducing proinflammatory and immune-evasion mechanisms that further promote SCC progression (15). SMAD4 loss or suppression may cooperate with reduced ERK signaling to increase SOX2 expression and promote a squamous phenotype. In Smad4fl/fl;KrasG12D mice, inhibition of ERK1/2 resulted in widespread formation of lung SCC (16). SMAD4 can also suppress SOX2 through a noncanonical mechanism involving three-dimensional genome regulation (17). By sequestering the chromatin regulator EP300, SMAD4 prevents its enrichment at SOX2 enhancers and promoter regions, restricts formation of SOX2-associated chromatin loops, and suppresses SOX2 transcription. Notably, SMAD4 loss in the breast has been reported to induce AST, while isolated SMAD4 knockout can drive spontaneous SCC formation in mouse skin, oral cavity, and stomach (18).

The alteration identified in this case was SMAD4 p.D351V, rather than SMAD4 gene deletion, and it was classified as a class III variant of uncertain significance under ACMG criteria. Its effect on SMAD4 function therefore warrants further investigation. A recent functional study showed that SMAD4 p.D351V can impair SMAD-dependent BMP/TGF-β signal transduction and affect the normal interactions of SMAD4 with SMAD1/SMAD3 (19). Given previous evidence linking SMAD4 dysfunction to squamous cell carcinoma formation, the potential impact of SMAD4 p.D351V on these signaling functions provides a theoretical basis for a possible biological association with AST in this case. However, whether this variant also affects the noncanonical functions of SMAD4, and whether it can directly promote squamous lineage transdifferentiation in the context of EGFR-mutant lung adenocarcinoma, remain to be determined.

Unlike SMAD4 p.D351V, ATM p.E1139K has not been directly shown to impair ATM activity. ATM is a key regulator of the DNA damage response and also contributes to cellular redox homeostasis. Experimental evidence links loss of ATM function to increased reactive oxygen species (ROS) accumulation and mitochondrial dysfunction (20). Separately, studies of AST have shown that excessive ROS accumulation can suppress Wnt signaling through FOXO3A and disrupt the balance between adenocarcinoma and squamous lineage programs (21). However, these findings do not establish a direct mechanistic link between ATM deficiency and AST, nor do they demonstrate that ATM p.E1139K causes loss of ATM function. Thus, whether ATM p.E1139K affects ATM function or contributes to lineage plasticity in this case requires further functional investigation.

### 3.2 Clinical implications

#### 3.2.1 Diagnostic considerations

During disease progression, the patient developed pleural metastases and a small pleural effusion. Subsequent plasma-based NGS did not identify clinically relevant variants that could explain resistance or guide subsequent treatment, raising the question of whether pleural effusion could provide additional value for molecular assessment. Percutaneous closed pleural brushing combined with cell-block preparation has been reported to improve the diagnostic yield for malignant pleural effusion, suggesting that this approach may provide a higher tumor-cell yield than conventional pleural sampling (27). Importantly, independent studies have shown that cell-free DNA from pleural effusion supernatant is a reliable substrate for targeted NGS in NSCLC and can provide complementary mutation detection to blood-based testing, with higher detection rates in some settings (28). Therefore, in patients with pleural progression and pleural effusion, particularly when plasma NGS is uninformative, pleural effusion may serve as a useful alternative source for molecular profiling and may inform subsequent treatment decisions (28–30).

#### 3.2.2 Treatment considerations

After HT, the patient received nab-paclitaxel, carboplatin, and tislelizumab and achieved a partial response at 5 months later. During treatment, no serious adverse events or immune-related adverse events were observed. Evidence to guide treatment of AST remains limited. For patients with an EGFR T790M mutation, osimertinib may be a treatment option worth considering (22). The AURA3 trial demonstrated that osimertinib significantly prolonged progression-free survival compared with standard chemotherapy in patients with EGFR T790M-positive NSCLC (23). However, AURA3 was not designed specifically for patients with AST, and evidence for osimertinib after AST in patients harboring EGFR T790M is currently limited to case reports; its efficacy in this setting requires further validation. For patients without T790M, one case report described prolonged survival with afatinib (24). In many cases, however, expert consensus supports the use of chemotherapy regimens for squamous cell carcinoma, while immunotherapy should be evaluated in clinical trials (25). Some studies suggest that immune checkpoint inhibitors may have limited efficacy in AST. In one study of 21 patients with EGFR-mutant lung cancer who developed HT, the median progression-free survival (mPFS) with immunotherapy was 1.6 months (2). However, that study did not examine the association between PD-L1 expression and treatment response or further stratify patients by HT subtype, which may limit interpretation of the mPFS estimate for AST specifically. Immunotherapy efficacy may, in fact, be associated with PD-L1 expression. One report described a patient with EGFR L858R-mutant lung adenocarcinoma who developed squamous transformation after osimertinib. PD-L1 expression increased substantially after transformation (90% vs. 1% before transformation), and the patient achieved a durable response lasting 47 months with pembrolizumab monotherapy (26). This case suggests that immunotherapy may benefit selected patients with AST and high PD-L1 expression. Overall, the optimal treatment strategy remains uncertain and should take into account factors such as EGFR T790M status and PD-L1 expression. Prospective controlled studies are needed to define the best treatment approach.

### 3.3 Limitations

Although SMAD4 p.D351V and ATM p.E1139K were newly detected in the post-transformation tumor, this case cannot establish a causal relationship between these alterations and HT. SMAD4 p.D351V may affect core SMAD4 functions, and SMAD4 loss has been implicated in SCC development; however, any association between this variant and AST in this patient remains hypothetical. The functional consequences of ATM p.E1139K are also uncertain. These alterations should therefore be regarded as candidate molecular events that may be involved in AST rather than established drivers, and their biological significance requires validation in functional studies and larger longitudinal cohorts. In addition, because the original surgical slides were unavailable for re-review, the possibility of a minor squamous subclone that was not sampled at baseline cannot be completely excluded, despite the original pathology report showing no SCC or adenosquamous carcinoma component. Accordingly, the histologic evolution in this case is best described as clinically and pathologically consistent with AST, while selective expansion of a pre-existing subclone remains a possible alternative explanation.

## 4 Conclusion

We report a case of AST after multiple lines of therapy in a patient with EGFR-mutant lung adenocarcinoma and use longitudinal NGS to characterize molecular changes accompanying transformation. The SMAD4 p.D351V and ATM p.E1139K variants were newly detected in the post-transformation tumor. The potential functional impact of SMAD4 p.D351V provides a theoretical basis for a possible biological association with AST, whereas the functional significance of ATM p.E1139K remains unclear. Whether either variant contributes to tumor lineage transformation requires further investigation. After transformation, the patient received carboplatin, nab-paclitaxel, and tislelizumab and achieved a partial response. This observation suggests that platinum–taxane-based chemotherapy combined with immunotherapy may be considered in selected clinical contexts, but the contribution of each treatment component cannot be determined from this single case. Further studies are needed to clarify the molecular mechanisms of AST and to identify optimal treatment strategies after transformation.

## Data Availability

The original contributions presented in the study are included in the article/supplementary material. Further inquiries can be directed to the corresponding authors.
